# Efficacy and safety of biologics in primary sclerosing cholangitis with inflammatory bowel disease: A systematic review and meta-analysis

**DOI:** 10.1097/HC9.0000000000000347

**Published:** 2024-01-11

**Authors:** Ayesha Shah, Michael P. Jones, Gavin Callaghan, Thomas Fairlie, Xiaomin Ma, Emma L. Culver, Katherine Stuart, Peter De Cruz, James O’Beirne, James H. Tabibian, Axel Dignass, Ali Canbay, Gregory J. Gores, Gerald J. Holtmann

**Affiliations:** 1 The University of Queensland, Faculty of Medicine, Australia; 2 Department of Gastroenterology & Hepatology, Princess Alexandra Hospital; 3 Translational Research Institute, Queensland, Australia; 4 AGIRA (Australian Gastrointestinal Research Alliance) and the NHMRC Centre of Research Excellence in Digestive Health; 5 Department of Psychology, Macquarie University, Sydney, New South Wales, Australia; 6 Translational Gastroenterology Unit, Oxford University Hospitals NHS Foundation Trust, Oxford, United Kingdom; 7 NIHR Oxford Biomedical Research Centre, University of Oxford, Oxford, United Kingdom; 8 Department of Gastroenterology, Austin Health, Melbourne, Australia; 9 Department of Medicine, The University of Melbourne, Melbourne, Australia; 10 University of the Sunshine Coast, Sunshine Coast, Queensland, Australia; 11 Sunshine Coast University Hospital, Sunshine Coast, Queensland, Australia; 12 David Geffen School of Medicine at UCLA, Los Angeles, California, USA; 13 Department of Medicine I, Agaplesion Markus Hospital, Frankfurt, Germany; 14 Department of Medicine, University Hospital of the Ruhr-University Bochum, Germany; 15 Division of Gastroenterology and Hepatology, Mayo Clinic, Rochester, Minnesota, USA

## Abstract

**Background:**

Primary sclerosing cholangitis (PSC) is an immune-mediated, chronic cholestatic liver disease. Currently, liver transplantation is the only established life-saving treatment. Several studies have evaluated the effect of different biologic therapies on PSC with inconclusive findings. We conducted a systematic review and meta-analysis to assess the effects of biologics in PSC and associated inflammatory bowel disease (IBD).

**Methods:**

MEDLINE, Scopus, and Embase were searched up to July 31, 2023, for studies reporting the effects of biologics in patients with PSC-IBD. Effects of biologic therapy on alkaline phosphatase, total bilirubin, ulcerative colitis response score, and adverse events were calculated and expressed as standardized difference of means (SMD), proportions, and 95% CI using a random-effects model.

**Results:**

Six studies, including 411 PSC-IBD patients who received biologics, were included. Biologic treatment was associated with no change in alkaline phosphatase (SMD: 0.1, 95% CI: −0.07 −0.17, *p*=0.43), but a small and statistically significant increase in total bilirubin (SMD: 0.2, 95% CI: 0.05–0.35, *p*<0.01). 31.2% (95% CI: 23.8–39.7) of patients with IBD achieved endoscopic response, and there was a significant improvement in ulcerative colitis response score (SMD: −0.6,95% CI: −0.88 to 0.36, *p*<0.01). Furthermore, 17.6% (95% CI: 13.0–23.5) of patients experienced adverse events severe enough to discontinue therapy, and 29.9% (95% CI: 25.2–34.8) had a loss of response to biologics.

**Conclusions:**

Treatment of patients with PSC-IBD with biologics (vedolizumab, infliximab, and adalimumab) was not associated with improvement of biochemical markers of cholestasis. Biologics are effective in treating the colitis associated with PSC. Vedolizumab was associated with worsening liver enzymes in contrast to other biologics, a finding that warrants further study.

## INTRODUCTION

Primary sclerosing cholangitis (PSC) is a rare, immune-mediated, chronic cholestatic liver disease characterized by inflammation and fibrosis of intrahepatic and extrahepatic bile ducts, leading to progressive fibrotic transformation of bile ducts, resulting in the development of multifocal bile duct strictures.^1^ The natural history of PSC is dominated by the risk of progressive biliary strictures, which can lead to cholangitis, biliary cirrhosis, end-stage liver disease, and an increased risk of developing digestive neoplasia.^1^ Typically, PSC is associated with a unique phenotype of inflammatory bowel disease (IBD) characterized by pancolitis, with right-sided predominance, rectal sparing, and an increased risk of colorectal cancer.^2^

Currently, there is no effective medical therapy that has been shown to cure or halt PSC disease progression, and liver transplantation (LT) represents the only curative option.^3^ Despite being an immune-mediated disease, immunosuppressive agents, including corticosteroids, tacrolimus, cyclosporine, azathioprine, methotrexate, and penicillamine, have been shown to have no or minimal clinical benefit in PSC.^3^

Although the pathogenesis of PSC is not fully understood, the close association with IBD has resulted in therapies exploring the bidirectional interplay of the gut-liver axis^4^ with the assumption that IBD therapies might be beneficial in PSC. Three biologics (infliximab, adalimumab which are chimeric and humanized anti-TNF alpha antibodies, respectively, and vedolizumab which is a fully humanized monoclonal antibody, an “integrin antagonist,” which binds to α4β7 integrin expressed on T-lymphocytes) have been successfully used in treating Crohn disease and ulcerative colitis (UC)^5^ have also been evaluated in PSC (mainly to treat the associated colitis). TNF alpha has been suggested to play a central role in the immune responses of liver damage in PSC;^6^ thus, inhibition of the common end inflammatory molecule, TNF alpha is considered potentially effective in patients with PSC and associated IBD.

In theory, vedolizumab, by inhibiting the adhesion and migration of leukocytes into the gastrointestinal tract^7^ may be an attractive option for PSC, as it blocks gut-homing lymphocyte trafficking to bile ducts which have been linked with the pathophysiology of PSC.^8^ Although it is acknowledged that several biologics other than anti-TNF and anti-integrins have been approved for use in IBD,^9^ data regarding their use in PSC-IBD are sparse (often reported in case reports or case series or only one cohort study) hence were excluded from this meta-analysis.

Although biologics have been extensively studied in IBD, the data in PSC are limited due to a small number of studies with small sample sizes. We thus conducted a systematic review and meta-analysis to determine and compare the efficacy and safety of infliximab, adalimumab, and vedolizumab for the treatment of patients with PSC with or without concomitant IBD. The primary end point was change from baseline in the markers of cholestasis [alkaline phosphatase (ALP) and total bilirubin] postbiologic therapy. The secondary end points were (1) change from baseline in other liver enzymes including alanine transaminase (ALT) and aspartate transaminase (AST) postbiologic therapy, (2) change from baseline in markers of IBD activity including the proportion of patients with colitis who achieved endoscopic and clinical response (improvement) or remission, (3) adverse events (AEs) leading to discontinuation of biologics or loss of response to biologics and, (4) proportion of patients who developed liver-related events during the follow-up period.

## METHODS

### Protocol and registration

This systematic review and meta-analysis meet the Preferred Reporting Items for Systematic Reviews and Meta-analysis (PRISMA) statement requirements.^10,11^ The protocol for this Systematic Review was prospectively registered with PROSPERO (CRD42022379629).

### Search strategy

**FIGURE 1:**
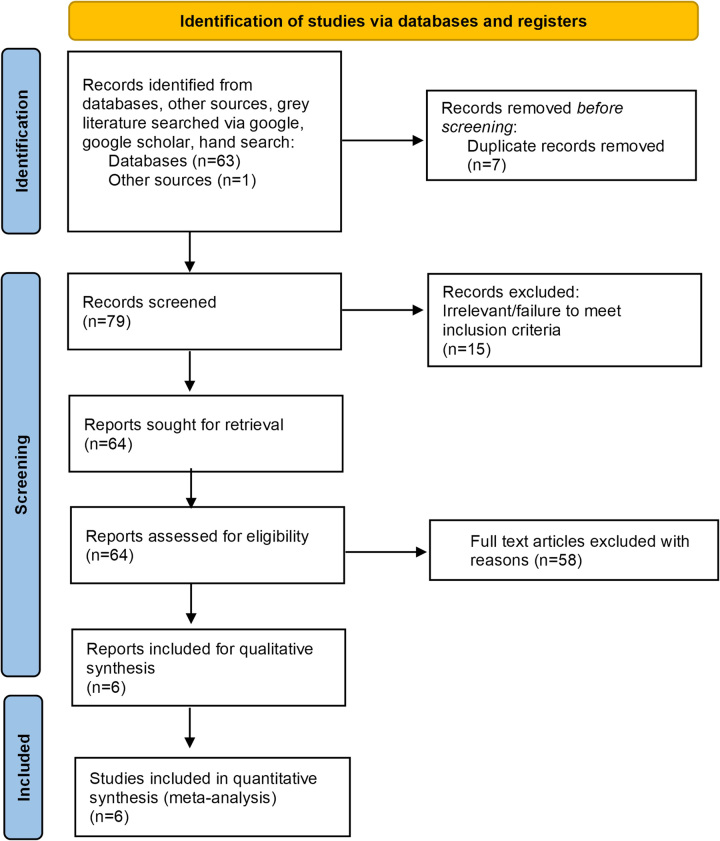
Preferred Reporting Items for Systematic Reviews and Meta-Analyses (PRISMA) flow diagram.

Electronic databases, including PUBMED, MEDLINE (OvidSP), SCOPUS, and EMBASE, were searched from initiation (1966) up to July 31 2023, of all studies assessing the use of biologics in patients with PSC with or without IBD. The detailed literature search strategy is outlined in the PRISMA flow diagram (Figure 1) and Supplemental Figure S1, http://links.lww.com/HC9/A711 and was conducted with the expert assistance of our librarian. Further details have been outlined in the Supplemental File, http://links.lww.com/HC9/A711.

### Selection of studies

Two authors (Ayesha Shah and Gavin Callaghan) independently conducted an initial screen of abstracts and titles. Abstracts were eliminated in this initial screening if they were case reports or case series, animal studies, or if they did not investigate the association between biologic therapy and PSC with or without IBD. Full texts of the remaining articles were retrieved and reviewed.

Articles were considered for inclusion only if they reported original data from open-labeled observational studies or randomized controlled trials (RCTs), reported use of biologics (infliximab, adalimumab, or vedolizumab) per standard dosing scheduled for the treatment of patients with an established diagnosis of IBD with concurrent PSC, and the manuscript or abstracts were published in peer-reviewed journals. The diagnosis of PSC was established by the following criteria: cholestatic liver biochemistry or a raised liver ALP for at least 6 months with cholangiographic (eg, magnetic resonance cholangiography, endoscopic retrograde cholangiography, and percutaneous transhepatic cholangiography) evidence of characteristic bile duct changes with multifocal strictures and segmental dilatations, and exclusion of secondary causes of sclerosing cholangitis.^3^ We excluded studies in which participants were aged below 16 years and studies where data could not be extracted. Eligibility criteria for study inclusion are provided in Supplemental Table S1, http://links.lww.com/HC9/A711. The studies that were excluded are outlined in Supplemental Table S2, http://links.lww.com/HC9/A711. Disagreements between reviewers were resolved by mutual consensus after reference to the original published article.

### Data extraction and quality assessment

All data were extracted independently by 2 authors (Ayesha Shah and Gavin Callaghan) into a Microsoft Excel spreadsheet (Office 360; Microsoft Corp, Redmond, WA), with disagreements resolved by consensus. The variables extracted are detailed in the Supplemental File, Materials and Methods,http://links.lww.com/HC9/A711. The quality of the studies was assessed using the Joanna Briggs Institute (JBI) critical appraisal tools,^12^ outlined in detail in the Supplemental Materials and Methods section, http://links.lww.com/HC9/A711.

### Data analysis

Data were recorded as mean and SD. Median values and ranges were transformed to mean and SD.^13^ In an initial step, case numbers of patients with PSC-IBD treated with different biologic therapies were determined. In a second step, changes from the baseline values in ALP, total bilirubin, ALT, AST, and the UC response score postbiologic therapy were calculated. Further details are provided in the Supplemental File, Materials and Methods, http://links.lww.com/HC9/A711.

The standardized difference in means and 95% CI for the primary outcome measures (ALP and total bilirubin) and secondary outcome measures (ALT, AST, and UC response score) were calculated from the prebiologic and postbiologic therapy. Subgroup analyses according to the type of biologic (vedolizumab, infliximab, and adalimumab) on each primary and secondary outcome measures were also carried out. Pooled discontinuation rates due to AEs and loss of response to biologic were calculated. Lastly, pooled rates of patients with IBD who attained response or remission (endoscopic or clinical) and liver-related adverse events were calculated. Proportions and 95% CI were calculated when appropriate. Finally, we considered undertaking sensitivity analyses but decided that they were not warranted due to the high quality of studies included in this meta-analysis.

Analyses were carried out utilizing the comprehensive Meta-analysis software (CMS) Version 3.3.070., NJ, USA. In the results section, we report the observed (unweighted) number of positive cases and total tested in addition to the weighted pooled estimates of response rates. Standardized mean difference and pooled response rates were calculated using a random-effects model^14^ to appropriately account for between-study variability. A standardized mean difference (SMD) can be interpreted as a change in SD units and values >0.5 are considered moderate and >0.8 are considered large. The statistical package CMS used logit transformation of proportions (response rates) and the variance of the logit to estimate pooled event rates both within groups and in comparing event rates between groups. If any numerator of a response rate had a value of 0, then the CMS software automatically adds a fixed value of 0.5 to the respective cell to allow computation of the variance of the log odds and log OR. Between-study variation was evaluated using Cochrane test^15^ and was quantified through the *I* ^2^ index in which values close to 100 indicate substantial variation between studies while values close to zero indicate minimal between-study variation. Standard approaches (Egger test^16^ and inspection of Funnel plots) were applied to identify potential publication biases. Further, either chi-squared test *p*<0.10 or *I*² >50% indicated substantial heterogeneity.

## RESULTS

### Selection outcome

The initial literature search revealed 62 publications, and of these, 39 published articles addressed the study question and were retrieved for further evaluation. Thirty-one articles were excluded due to not fulfilling the inclusion criteria leaving 6 eligible studies (Figure 1).

The final data set included eight studies with 411 patients with PSC-IBD who were treated with biologics (infliximab, adalimumab, and vedolizumab). All studies assessed the impact of biologics on IBD in patients with PSC. One RCT^17^ compared the efficacy of placebo against infliximab, 3 cohort studies^18–20^ assessed the safety and efficacy of vedolizumab, one cohort study^21^ assessed and compared the efficacy and safety of adalimumab and infliximab and 1 cohort study^22^ assessed and compared the efficacy and safety of vedolizumab, infliximab and adalimumab in patients with PSC-IBD. The primary and secondary outcomes, demographic and clinical characteristics of the patients included in the 6 studies are outlined in Tables 1 and 2, respectively. The inclusion and exclusion criteria for each of the 6 studies are outlined in Supplemental Table S3, http://links.lww.com/HC9/A711. The findings of this meta-analysis are summarized in Supplemental Table S4, http://links.lww.com/HC9/A711.

**TABLE 1:** Summary of the results of biologic therapy in patients with primary sclerosing cholangitis

						IBD			Delta postbiologic therapy (baseline value – post-treatment values)							Loss of response	
No	References	Study type	Biologic type	Treatment duration (d)	N	UC	CD	IBD-U	ALP	*p*	Total bilirubin	*p*	UC Mayo Score	*p*	AEs leading to discontinuation of biologic	Primary or Secondary	Proportion of IBD patients with endoscopic response to biologic therapy, n/N (%)
1.	Lynch et al^18^	Cohort study	Vedolizumab	412	102	66	30	6	−39.6	0.028	−2.8	<0.01	+0.8^c^	<0.01	6	32	42/74 (56.8)
2.	Caron et al^19^	Cohort study	Vedolizumab	210	54	33	21	0	−11	0.66	−10	0.51	+2^d^	<0.01	19	14	14/34 (41.2)
3.	Christensen et al^20^	Cohort study	Vedolizumab	252	26	11	14	0	−43	0.99	−2.8	0.96	NA	NA	1	6	4/13 (40)
4.	Tse et al^22^	Cohort study	Vedolizumab	224	27	16	10	1	−50	0.11	12	0.46	NA	NA	NA	NA	NA
			Infliximab	224	42	25	15	2	−37	0.23	−3.43	0.06	NA	NA	NA	NA	NA
			Adalimumab	224	19	14	5	0	70	0.00	−1.71	0.06	NA	NA	NA	NA	NA
5.	Hedin et al^21^	Cohort study	Infliximab	336	110	68	38	4	18.3	0.31	−1.6	<0.01	NA	NA	26	49	22/95 (23.2)
		Cohort study	Adalimumab	336	31	16	14	1	40.3	<0.01	1.1	0.65	NA	NA	8	9	NA
6.	Hommes et al^17^	RCT^a^	Infliximab	84	6	NA	NA	NA	−40	n.s	NA	NA	NA	NA	0	NA	NA

a Indicates prospective studies; UC activity was measured using.

b Mayo endoscopic subscore.

c Partial Mayo Clinic Score and.

d Combined endoscopic response rates were available with no separate data for Infliximab and Adalimumab.

Abbreviations: AEs, adverse events; ALP, Alkaline phosphatase; CD, Crohn disease; IBD-U, inflammatory bowel disease unclassified; NA, not applicable; n.s., not significant; RCT, randomized controlled trial; UC, ulcerative colitis.

**TABLE 2:** Characteristics of the studies included in the systematic review and meta-analysis

						Type of PSC							
No	References	Year	Country	Median age (Yrs)	Sex male n (%)	Large duct n	Small duct n	Overlap n	Cirrhosis, n (%)	Post-transplant PSC, n (%)	Primary end point (improvement/normalization at the end of treatment)	Secondary end point (improvement/normalization at the end of treatment)	Other Meds
1.	Lynch et al^18^	2019	Europe and North America multicenter	31.4 (14.2)^a^	64 (62.8)	92	8	2	21 (20.6)	0	Change in ALP, ALT, AST, and bilirubin levels at baseline, week 6 (ie, day 42), week 14 (ie, day 96), and last follow-up while on vedolizumab. Proportion of patients whose ALP dropped by ≥20% from baseline to the last follow-up	Other end points included response of IBD to treatment (improved, unchanged, or worsened, judged by the treating clinician, as well as endoscopic score) and liver-related outcomes	61 patients were on concomitant UDCA
2.	Caron et al^19^	2019	Europe multicenter	24.9 (18.0–34.6)	37 (68.5)	NA	NA	NA	NA	0	Decrease in the ALP level of at least 50% from baseline to week 30 or 54	A change in any liver enzyme levels and an assessment of the efficacy and safety of vedolizumab in IBD	45 patients were on UDCA, 26 on steroids, and 18 on other immunosuppressants
3.	Christensen et al^20^	2018	Australia and North America multicenter	24 (20–29)	NA	NA	NA	NA	2	3	Decrease in ALP level at weeks 14 and 30 in those with active PSC	Changes in total bilirubin, Mayo PSC Risk Score, ALT, AST from baseline to weeks 14 and 30 in those with active PSC, clinical outcomes for the bowel and the development of adverse events	7 patients were on UDCA
4.	Tse et al^22^	2018	USA	NA	55	NA	NA	NA	NA	NA	Hepatic biochemistries were abstracted ≤3 months before and 6–8 and 12–14 months after biological initiation or after PSC diagnosis, whichever occurred later	Radiologic assessment of biliary stenoses and hepatic stiffness were compared anytime within 12 months before and 6-12 months after biological initiation as obtained by means of abdominal ultrasound, abdominal MRI, or magnetic resonance (MR) elastography	19 patients were on UDCA, 25 on corticosteroids, and 30 on immunomodulators
5.	Hedin et al.^21^	2020	Europe and North America multicenter	NA	89	NA	NA	NA	18	0	Data on the level of ALP were collected	IBD response was defined as either endoscopic response or, if no endoscopic data were available, clinical response, as determined by the treating clinician or measurements of fecal calprotectin. Remission was defined more stringently as endoscopic mucosal healing	59 patients were on UDCA, 71 on mesalazine, 73 on corticosteroids, and 63 on immunomodulators
6.	Hommes et al^17^	2008	Netherlands	42 (11)^a^	4	NA	NA	NA	0	0	Decrease in the ALP level of at least 50% from baseline to Week 18	A blinded histologic assessment of liver biopsy samples, obtained at weeks 0 and 26, was performed using a predefined scoring system, including scores for inflammation, fibrosis, and cholestasis	3/6 were on mesalazine and 5/6 were on UDCA

a Values expressed as mean (SD).

Abbreviations: ALP, alkaline phosphatase; ALT, alanine aminotransferase; AST, aspartate aminotransferase; IBD, inflammatory bowel disease; 6-MP, mercaptopurine; MR, magnetic resonance; NA, not applicable; PSC, primary sclerosing cholangitis; RCT, randomized controlled trial; UDCA, ursodeoxycholic acid.

### Effect of biologic therapy on primary outcome measures in patients with PSC-IBD

**FIGURE 2:**
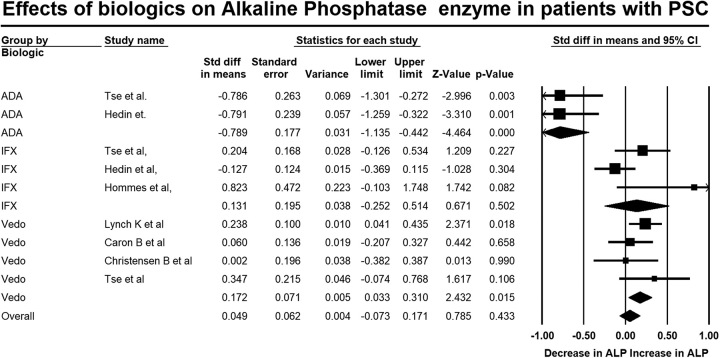
Forest plot of studies showing the change in ALP postbiologic therapy in patients with PSC and inflammatory bowel disease (standardized difference of means: 0.05, 95% CI: −0.07 to 0.17, *p*=0.43) (*I* ^2^=76.1, *p*<0.01). Abbreviations: ADA, Adalimumab; ALP, alkaline phosphatase; IFX, Infliximab; PSC, primary sclerosing cholangitis; Vedo, Vedolizumab.

**FIGURE 3:**
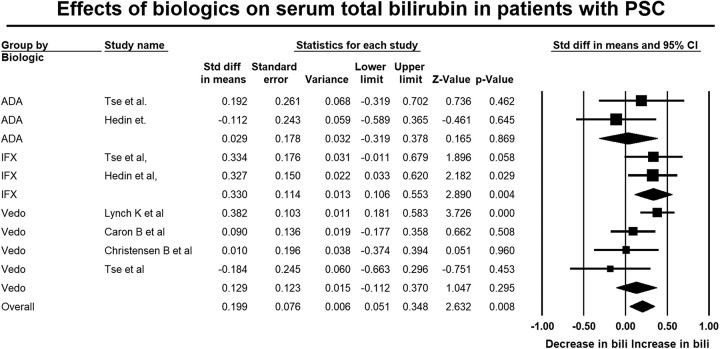
Forest plot of studies showing the change in total bilirubin postbiologic therapy in patients with PSC and inflammatory bowel disease (standardized difference of means: 0.2, 95% CI: 0.05–0.35, *p*=0.01) (*I* ^2^=30.5, *p*=0.18). Abbreviation: ADA, Adalimumab; IFX, Infliximab; PSC, primary sclerosing cholangitis; Vedo, Vedolizumab.

A total of 205 patients with PSC-IBD were treated with vedolizumab, 108 with infliximab, and 42 with adalimumab. Overall, biologic treatment was associated with no significant change in ALP level (SMD: 0.05, 95% CI: −0.07 to 0.17, *p*=0.43, Figure 2). There was a small but statistically significant increase in total bilirubin level (SMD: 0.20, 95% CI: 0.05–0.35, *p*=0.01, Figure 3). Substantial heterogeneity was seen in the analysis reporting on ALP (*I* ^2^=76.1, *p*<0.01) and moderate heterogeneity in the analysis reporting on total bilirubin (*I* ^2^=30.5, *p*=0.18).

### Influence of risk of bias on the effect of biologic therapy on primary outcome measures in patients with PSC-IBD

All studies included in this meta-analysis were deemed to be of high quality, as assessed by the JBI critical appraisal tool (Supplemental Table S5, http://links.lww.com/HC9/A711). Consequently, we did not conduct a sensitivity analysis based on the quality of the studies.

### Effect of biologic therapy on secondary outcome measures in patients with PSC-IBD: Effect of biologic therapy on other liver enzymes and PSC Mayo risk score

Four studies^18–20,22^ reported AST and ALT levels before and after treatment with biologics. Treatment with biologics was associated with no significant changes in AST (SMD: 0.15, 95% CI: −0.03 to 0.33, *p*=0.09, Supplemental Figure S2, http://links.lww.com/HC9/A711) or ALT (SMD: 0.06, 95% CI: −0.09 to 0.22, *p*=0.44, Supplemental Figure S3, http://links.lww.com/HC9/A711), with moderate heterogeneity in both analyses.

Only 1 out of the 6 studies^20^ included in this meta-analysis reported on PSC Mayo Risk Score and found that treatment with vedolizumab was not associated with improvement in mean Mayo PSC Risk Score, −0.40 (95% CI: −0.85 to 0.05) at baseline versus −0.38 (95% CI: −0.83 to 0.08) at week 30, *p*=0.90).

### Effects of individual biologic therapies

Treatment with adalimumab was associated with a large and statistically significant reduction in ALP (SMD: -0.79, 95% CI: −1.14, −0.44, *p*<0.01, Figure 2), but no significant changes were seen in total bilirubin (SMD: 0.03, 95% CI: −0.32, 0.38, *p*=0.87, Figure 3), AST (SMD: 0.03, 95% CI: −0.43 to 0.50, *p*=0.89, Supplemental Figure S2, http://links.lww.com/HC9/A711), or ALT (SMD: −0.13, 95% CI: −0.58 to 0.32, *p*=0.56, Supplemental Figure S3, http://links.lww.com/HC9/A711).

On the other hand, treatment with vedolizumab was associated with a small but statistically significant increase in ALP (SMD: 0.17, 95% CI: 0.03–0.31, *p*=0.02, Figure 2), and no significant changes in AST (SMD: 0.13, 95% CI: −0.10 to 0.36, *p*=0.28, Supplemental Figure S2, http://links.lww.com/HC9/A711), ALT levels (SMD:0.11, 95% CI: −0.08 to 0.31, *p*=0.26, Supplemental Figure S3, http://links.lww.com/HC9/A711), or total bilirubin (SMD: 0.13, 95% CI: −0.11, 0.37 *p*=0.30, Figure 3) were observed.

Finally, treatment with infliximab did not result in significant changes in ALP (SMD: 0.13, 95% CI: −0.25,0.51, *p*=0.50, Figure 2) or ALT (SMD: 0.02, 95% CI: −0.31 to 0.36, *p*=0.90, Supplemental Figure S3, http://links.lww.com/HC9/A711) but a small increase in AST (SMD: 0.26, 95% CI: −0.08, 0.60, *p*=0.13, Supplemental Figure S2, http://links.lww.com/HC9/A711). However, treatment with infliximab was associated with a moderate and statistically significant increase in total bilirubin (SMD: 0.33, 95% CI: 0.11–0.55 *p*<0.01, Figure 3).

### Effect of biologic therapy on the colitis associated with PSC

**FIGURE 4:**
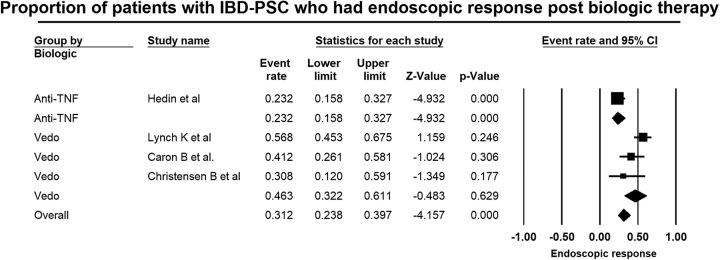
Forest plot of studies showing the proportion of patients with PSC and associated IBD, who achieved endoscopic response of their associated colitis after treatment with biologic therapy, 31.2% (95% CI: 23.8–39.7) (*I* ^2^=84.5, *p*<0.01). Abbreviations: IBD, inflammatory bowel disease; PSC, primary sclerosing cholangitis.

Four studies^18–21^ reported on endoscopic response, and 3 studies^19–21^ reported on clinical response from baseline to postbiologic therapy in patients with PSC-IBD. Overall, 31.2% (95% CI: 23.8–39.7) of patients with PSC-IBD had an endoscopic response postbiologic therapy. There was substantial heterogeneity in the overall analysis (*I* ^2^=84.5, *p*<0.01). The proportion of patients with endoscopic response was significantly higher with vedolizumab (46.3%, 95% CI: 32.2–61.1) compared to that observed with anti-TNF alpha inhibitors (23.2%, 95% CI: 15.8–32.7, Figure 4).

Furthermore, 47% (95% CI: 39.6–54.5) of patients with PSC-IBD achieved clinical response post-biologic therapy, with similar response rates for vedolizumab and anti-TNF alpha inhibitors (data not shown). The 2 studies^18,19^ reporting on UC clinical indices at baseline and postbiologic therapy showed a significant improvement in the UC response scores postbiologic therapy (SMD: −0.62, 95% CI: −0.88, −0.36, *p*<0.01, Supplemental Figure S4, http://links.lww.com/HC9/A711), with minimal heterogeneity in the analysis (*I* ^2^=0, *p*=0.94).

### Safety outcomes: Adverse events leading to discontinuation of biologic therapy

Four^18–21^ out of the 6 studies reported on AEs that were severe enough to discontinue biologic therapy (Table 1). Overall, 17.6% (95% CI: 13.0–23.5) of patients with PSC-IBD reported AEs leading to discontinuation of biologics, with considerable heterogeneity in the analysis (*I* ^2^=79.7, *p*<0.01), Supplemental Figure S5, http://links.lww.com/HC9/A711. Among the biologics, the incidence of AEs was highest for adalimumab at 20.5% (95% CI: 9.3–36.5) followed by infliximab at 17.7% (95% CI: 11.9–24.3) and lowest for vedolizumab at 9.8% (95% CI: 2.5–31.8).

### Loss of response leading to discontinuation of biologic therapy

Four studies^18–21^ reported on loss of response to biologic therapy. Overall, 29.9% (95% CI: 25.2–34.8) of patients with PSC-IBD treated with biologics had either primary or secondary loss of response during the follow-up period. There were no differences in loss of response rates between vedolizumab (28.6%, 95% CI: 22.1–35.7) and the anti-TNF alpha inhibitors (31.2%, 95% CI: 24.6–38.4, *p*=0.83).

### Risk of digestive neoplasia in patients with PSC-IBD treated with biologics

Two studies^19,21^ with 240 patients with PSC-IBD reported on risk of digestive neoplasia with biologic therapy. Overall, 12/240 (5%, 95% CI: 2.6–8.6) patients with PSC-IBD treated with biologics developed digestive neoplasia. Among the biologics, the risk for digestive neoplasia was significantly higher for those treated with vedolizumab, 9/54 (16.7%, 95% CI: 7.9–29.3) followed by infliximab 3/147 (2.0%, 95% CI: 0.4–5.9, *p*<0.01). None of the patients with PSC-IBD treated with adalimumab developed digestive neoplasia during the follow-up period.

### Risk of liver-related adverse events in patients with PSC-IBD treated with biologics

Three studies^18,20,21^ reported on liver-related adverse events in 314 patients with PSC-IBD treated with biologics during the follow-up period. Thirty-three (10.5%, 95% CI: 7.4–14.4) patients experienced a liver-related adverse event during follow-up, 9 (2.9%, 95% CI: 1.3–5.4) patients underwent LT, 10 (3.2%, 95% CI: 1.5–5.8) patients had at least 1 episode of cholangitis, 6 (1.9%, 95% CI: 0.7–4.1) patients had new onset ascites, 5 (1.6%, 95% CI: 0.5–3.6) patients developed jaundice, and 2 (0.6%, 95% CI: 0.08–2.2) patients each were found to have a dominant stricture and biliary dysplasia.

### Effect of biologics on patients with PSC with established cirrhosis

Two studies^18,21^ reported on the effect of biologics in patients with PSC-IBD with cirrhosis. Hedin et al^21^ found that in 18 patients with PSC-IBD with cirrhosis, treated with anti-TNF alpha inhibitors, the median baseline ALP and total bilirubin levels did not change significantly over 6- or 12- month follow-up period. In contrast, Lynch et al^18^ showed that in the 21 patients with PSC-IBD and cirrhosis (as compared to those without cirrhosis) treated with vedolizumab, there was a significant decrease in ALP from baseline to follow-up of 20% (OR: 4.70; 95% CI: 1.61–13.76, *p*=0.01).

### Effect of biologics on IBD response in post-transplant patients with PSC

Only one study^21^ included post-liver transplant recipients with PSC-IBD. LT did not affect response to biologic therapy. In this study, LT did not affect the IBD response to biologic therapy compared to nontransplanted patients with PSC (4/7, 57.1%, 95% CI: 18.4–90.1 vs. 50/104, 48.1%, 95% CI: 38.2–58.1, respectively, *p*=0.78).

### Link between change in ALP and IBD response in patients with PSC on biologics

Although 4 studies^18–21^ reported on the link between positive IBD response and change in ALP, the data could not be extracted for conducting subgroup analyses. While 1 study^21^ found that a positive IBD response was significantly associated with a lower ALP level at the last follow-up, the other study^20^ failed to show a similar trend. With regard to vedolizumab, Lynch et al^18^ found normal ALP at baseline was associated with an endoscopic IBD response; however, Caron et al^19^ showed no impact of vedolizumab on clinical activity of IBD according to ALP level at baseline. Thus, the association between change in ALP and IBD response remains unclear.

## DISCUSSION

### Summary of findings

This is the first systematic review and meta-analysis focusing on the efficacy and safety of biologics in adult patients with PSC with associated IBD. While limited to retrospective multicenter cohort studies and one small RCT, the data suggest that biologics do not improve markers of cholestasis and other liver enzymes in patients with PSC-IBD. In contrast, biologics are effective in treating the colitis associated with PSC, while the IBD endoscopic response rates to biologics were lower in patients with PSC-IBD as compared to those reported in the literature for IBD without PSC.^23,24^ Moreover, the rate of AEs leading to discontinuation of biologics was higher in patients with PSC-IBD compared to those with IBD alone.^25^ Furthermore, patients with PSC-IBD had comparable rates of loss of response to biologic therapy to those seen in patients with IBD alone.^26,27^

### Comparison with previous research

Compared to the previously published cohort studies, this meta-analysis enables the comparison of the effects of different biologics in patients with PSC-IBD in terms of their clinical efficacy and adverse outcomes. First, we assessed the effect of individual biologics on liver enzymes in patients with PSC-IBD. Treatment with vedolizumab was associated with a small but statistically significant increase in ALP and a ( nonsignificant) increase in other liver enzymes, with no effect on total bilirubin. The incidence of liver enzyme abnormalities is similar in patients with IBD-only treated with vedolizumab compared to placebo (2.1/100 person years vs. 2.8/100 person years, respectively^28^). These findings may point toward the possibility that vedolizumab could adversely affect liver function and even accelerate the progression of PSC. However, these findings must be interpreted with caution as none of the studies included in this meta-analysis reported on reversibility of DILI after discontinuation of vedolizumab or evidence of liver injury on histology or progression of biliary disease on imaging.

Infliximab treatment had no effect on liver enzymes but was associated with a moderate and significant increase in total bilirubin level. Interestingly, adalimumab treatment was associated with a large improvement in ALP, but no effect was observed on other liver enzymes or total bilirubin. This isolated reduction in ALP during adalimumab treatment might be related to an effect on bone ALP, as TNF alpha has an important role in the regulation of bone homeostasis by means of activation of complex signaling pathways leading to gene transcription of several regulators of bone homeostasis.^29^

It is noteworthy that a significant proportion of patients with PSC-IBD (31.2%, 95% CI: 23.8–39.7) had an endoscopic IBD response, which was 2-fold higher for vedolizumab (46.3%, 95% CI: 32.2–61.1) compared to the anti-TNF alpha inhibitors (23.2%, 95% CI: 15.8–32.7, *p*<0.01). Moreover, there was a large and significant improvement in the UC response score, in patients with PSC-IBD treated with vedolizumab. The effectiveness of vedolizumab on mucosal inflammation in PSC-IBD was comparable to that observed in non-PSC-IBD cohorts;^23^ however, lower response rates were reported for anti-TNF alpha inhibitors in PSC-IBD compared to non-PSC-IBD cohorts.^24^

Overall, the rate of AEs leading to discontinuation of biologics in patients with PSC-IBD was higher (17.6%, 95% CI: 13.0–23.5) than that reported in the literature for non-PSC-UC cohorts (for infliximab 4% (95% CI: 1–14) and for vedolizumab 5% (95% CI: 3–7).^25^ Vedolizumab was associated with a lower drug discontinuation rate compared to the anti-TNF alpha inhibitors (9.8%, 95% CI: 2.5–31.8 vs. 18.3%, 95% CI: 13.4–24.5, *p*=0.03). The AE rates leading to discontinuation of vedolizumab treatment in patients with PSC-IBD were similar to those reported in the literature for patients with IBD alone treated with vedolizumab.^25^ Finally, the current meta-analysis reports similar loss of response rates to biologics among patients with PSC-IBD as compared to those reported in patients with IBD alone.^26,27^

In this systematic review and meta-analysis, approximately 10.5% (95% CI: 7.4–14.4) of patients with PSC-IBD treated with biologics had a liver-related adverse event, during the follow-up period (ranging from 274 to 561 d in the 3 studies reporting the data). Furthermore, treatment with biologics in patients with PSC and cirrhosis or after LT was not associated with any negative outcome in terms of their liver. Finally, 5% (95% CI: 2.6–8.6) patients with PSC-IBD treated with biologics developed gastrointestinal or hepatobiliary neoplasia with the risk being significantly greater for vedolizumab (after median follow-up of 1.6 y) as compared to that for infliximab with only 1-year follow-up after treatment. This is likely due to the small number of studies reporting liver-related adverse events, the low incidence of events, and the variable follow-up periods. However, it remains uncertain if these events reflect the natural history of PSC or they are a consequence of biologic therapy. Thus, the findings of these subgroup analyses must be interpreted with caution.

### Limitations

One major limitation of this systematic review and meta-analysis is that most of the studies included are retrospective observational studies. Due to the retrospective study design only a proportion of patients contributed to the analysis at each time point because data were not always available. Moreover, there was a lack of standardized approach for data collection and reporting across the studies. Furthermore, instead of clinically significant end points such as need for LT, development of cirrhosis or cancer incidence only markers of cholestasis were used as primary end points. ALP is considered as one of the mainstay markers of disease activity in PSC and often has been the most common primary end point in clinical trials of PSC.^30^ However, ALP has well-established limitations^31^ and its utility as a signal of efficacy is suboptimal as it can fluctuate. In addition, many studies included in this meta-analysis had only short and variable duration of biologic therapy and follow-up periods, and there is a paucity of data on the impact of biologics on the natural course of PSC. Another limitation is the moderate to high degree of heterogeneity scores and high risk of bias seen in the primary and majority of the subgroup analyses.

### Clinical implications

In conclusion, our systematic review and meta-analysis suggest that biologics in patients with PSC-IBD do not have any clinical benefit on the liver disease, with a potential negative signal seen for vedolizumab. However, these conclusions are based on the effect of vedolizumab only on liver biochemistry and not on long-term outcomes of PSC. Among the anti-TNF alpha inhibitors, it is intriguing that treatment with adalimumab (but not infliximab) was associated with an isolated improvement in ALP and warrants further exploration. Thus, while biologics are effective in treating the colitis associated with PSC, they should be used cautiously considering their effects on the liver in patients with PSC-IBD.

### Future research directions

Future prospective RCTs with long-term follow-up are required to explore the impact of different biologics on the natural history of PSC. This would also allow us to investigate if different patient subgroups within the PSC cohorts or if certain characteristics or biomarkers influence the effectiveness of biologics.

However, for rare diseases like PSC conducting RCTs can be challenging, or impractical, thus observational studies can be extremely valuable. There is a lack of consensus regarding validated surrogate end points to measure a therapeutic effect on disease progression in PSC. Thus, future observational studies need to combine multiple surrogate end points relevant to PSC. These would include serum biochemistries, primarily ALP and bilirubin, transient elastography or biopsy-proven fibrosis progression, and clinically relevant outcomes, such as occurrence of events such as death, LT, complications of cirrhosis, and carcinoma. Collectively, it appears that by combining and aligning multiple surrogate end points, future observational studies can provide a more comprehensive understanding of disease progression and the therapeutic effects on PSC.

## Supplementary Material
