# Supplementary material for: Efficacy and safety of biologics in primary sclerosing cholangitis with inflammatory bowel disease: A systematic review and meta-analysis
Source: Hepatol Commun. 2024 Jan 11;8(1):e0347. doi: 10.1097/HC9.0000000000000347 (PMC10786591; doi:10.1097/HC9.0000000000000347)
Supplement: Supplementary file 1 [file hc9-8-e0347-s001.docx]

**Supplementary file for the manuscript:** Efficacy and safety of biologics in Primary Sclerosing Cholangitis with Inflammatory Bowel Disease: A Systematic Review and Meta-Analysis.

**MATERIALS AND METHODS**

**Legend of Tables and Figures:**

**Legend of Figures:**

**Figure S1:** Search strategy for MEDLINE

**Figure S2:** Forest plot of studies showing the change in Aspartate transaminase enzyme (AST) post biologic therapy in patients with PSC, (SMD:0.15, 95%CI: -0.03-0.33, p=0.09), (I^2^=35.02, p=0.17).

**Figure S3:** Forest plot of studies showing the change in Alanine transaminase enzyme (ALT) post biologic therapy in patients with PSC, (SMD:0.06, 95%CI: -0.09-0.22, p=0.44), (I^2^=27.6, p=0.23).

**Figure S4:** Forest plot of studies showing the change in the ulcerative colitis (UC) response score post biologic therapy in patients with PSC (SMD: -0.62,95%CI: -0.88, -0.36, p<0.01), (I^2^=0, p=0.94).

**Figure S5:** Forrest plot of studies showing proportion of patients with PSC treated with biologics, who developed adverse events severe enough to discontinue treatment, 17.6% (95%CI:13.0-23.5), (I^2^=79.7, p<0.01).

**Legend of Tables:**

**Table S1**: Eligibility criteria for the studies included in systematic review and meta-analysis.

**Table S2:** Exclusion criteria for studies excluded from the systematic review and meta-analysis.

**Table S3:** Detailed inclusion and exclusion criteria for the studies included in this systematic review and meta-analysis.

**Table S4**: Summary of findings reported in the systematic review and meta-analysis.

**Table S5:** Joanna Briggs Institute (JBI) Critical Appraisal Tools for quality assessment of the studies included in the systematic review and meta-analysis.

**MATERIALS AND METHODS**

**Search strategy**

The initial search was not limited to specific languages to capture all appropriate studies. A further advanced search was conducted. Grey literature was searched with Google and Google Scholar, and the ‘Snowball’ method which refers to using the reference list of a paper or the citations to the paper to identify additional papers was utilised to identify all relevant articles.

**Data extraction and quality assessment**

During the data collection process, the following data was extracted from the studies: author, year of publication, journal, study design, country, pre and post treatment values for ALP, ALT, AST, total serum bilirubin, PSC Mayo risk score, full Mayo score and Mayo endoscopic sub score for UC, Harvey-Bradshaw index (HBI), Crohn's disease activity index (CDAI) and Simple Endoscopic Score for Crohn Disease (SES-CD) for CD. The extracted dataset also contained information on the gender distribution, proportion of patients with co-existing IBD, type of IBD, mean age, dose, type and duration of biologic therapy, concurrent use of other medications, total number of reported AEs, total number of AEs leading to discontinuation of biologic therapy, liver-related outcomes (including being listed for LT, development of cirrhosis, portal hypertension or complication of portal hypertension, biliary dysplasia, cholangitis and dominant strictures) during the follow up period, clinical criteria for diagnosis of PSC, type of PSC, presence of overlap with other autoimmune liver diseases; presence of cirrhosis and proportion of patients with PSC who had undergone LT and definition of primary and secondary outcomes.

IBD activity including, endoscopic and clinical response, and the proportion of IBD patients that achieved response or remission (both clinical and/or endoscopic) were recorded. IBD endoscopic response was defined as improvement or worsening of mucosal healing as judged by the treating clinician, and endoscopic scores at baseline and post-biologic therapy where available, namely the Mayo Endoscopic Subscore^1^, the Ulcerative Colitis Endoscopic Index of Severity^2^, for UC and the Simple Endoscopic Score for Crohn’s Disease^3^.

Where available, UC and CD clinical indices (henceforth referred to as UC or CD response scores in this meta-analysis) as determined by the treating clinician and where available, namely partial Mayo score for UC^4^, Harvey-Bradshaw index (HBI)^5^, Crohn's disease activity index (CDAI)^6^ for CD were also recorded. We also recorded proportion of IBD patients who were primary non-responders or had secondary loss of response to biologic therapy.

The quality of the studies was assessed using the Joanna Briggs Institute (JBI) critical appraisal tools^7^. The risk of bias was ranked as high when the study reached up to 49% of “yes” score, moderate when the study reached from 50 to 69% of “yes” score, and low when the study reached over 70% of “yes” score.

**Data analysis**

Data were recorded as mean and standard deviation. Median values and ranges were transformed to mean and SD^8^. Interquartile (IQR) or 5th and 95th percentile ranges were converted to SD through the following formula: SD = 0.7413 × (values at 75th percentile − values at 25th percentile) or SD = (values at 95th percentile − values at 5th percentile)/(2 × 1.645)^9^. To enable direct comparison, total bilirubin was measured as micromole per litre (μmol/L). To convert results from milligrams per decilitre (mg/dL) to μmol/L, multiply mg/dL by 17.1.

| **#1.** "Inflammatory Bowel"[ti] OR "IBD"[ti] OR "ulcerative colitis"[ti] OR "Colitis Gravis"[ti] OR "Crohn's"[ti] OR "Crohns"[ti]    **#2.** "sclerosing cholangitis"[ti]    **#3.** Vedolizumab OR Entyvio OR Vedolizumab OR Adalimumab OR Amjevita OR Cyltezo OR "D2E7 Antibody" OR Humira OR Hulio OR Halimatoz OR Cyltezo OR Amgevita OR Amsparity OR Adalimumab OR Infliximab OR Inflectra OR "Infliximab-abda" OR "Infliximab-dyyb" OR "MAb cA2" OR "Monoclonal Antibody cA2" OR Remicade OR Renflexis OR Inflectra OR Flixabi OR Infliximab    **#4**. #1 AND #2 AND #3    **#5.** Inflammatory Bowel Diseases[mh] OR Colitis, Ulcerative[mh] OR Crohn Disease[mh] OR Gastroenteritis[mh]    **#6.** Cholangitis, Sclerosing[mh]    **#7.** Cholangitis[mh]    **#8.** Sclerosis[mh]    **#9.** #7 AND #8    **#10.** #6 OR #9    **#11.** #5 AND #10 AND #3    **#12**. #4 OR #11 |
| --- |

**Figure S1:** Search strategy for MEDLINE

**Figure S2:** Forest plot of studies showing the change in Aspartate transaminase enzyme (AST) post biologic therapy in patients with PSC, (SMD:0.15, 95%CI: -0.03-0.33, p=0.09), (I^2^=35.02, p=0.17).

**Figure S3:** Forest plot of studies showing the change in Alanine transaminase enzyme (ALT) post biologic therapy in patients with PSC, (SMD:0.06, 95%CI: -0.09-0.22, p=0.440), (I^2^=27.6, p=0.23).

**Figure S4:** Forest plot of studies showing the change in the ulcerative colitis (UC) response score post biologic therapy in patients with PSC (SMD: -0.62,95%CI: -0.88, -0.36, p<0.01), (I^2^=0, p=0.94).

**Figure S5:** Forrest plot of studies showing proportion of patients with PSC treated with biologics, who developed adverse events severe enough to discontinue treatment, 17.6% (95%CI:13.0-23.5), (I^2^=79.7, p<0.01).

**Table S1:** Eligibility criteria for the studies included in systematic review and meta-analysis.

| **Eligibility criteria** |
| --- |
| - Adults (participants aged >16 years) - Randomized Controlled trials or Open labelled trials. - Patients with established diagnosis of PSC (utilizing clinically validated methods*) with concurrent IBD treated with infliximab, adalimumab, or vedolizumab. |
| * ALP greater than 1.5 times the upper limit of normal for at least 6 months with cholangiographic (e.g., magnetic resonance cholangiography, endoscopic retrograde cholangiography, percutaneous transhepatic cholangiography) evidence of characteristic bile duct changes with multifocal strictures and segmental dilatations, and exclusion of secondary causes of sclerosing cholangitis^10, 11^. |

**Table S2:** Studies excluded from the systematic review and meta-analysis.

Articles excluded, (n = 58)

**Reasons for exclusion:**

n = 10, case report^12-21^

n = 7, patients did not have PSC^22-28^

n = 5, unable to extract data^29-33^

n = 7, dual publication^34-40^

n = 25, review articles^41-65^

n = 1, no associated IBD^66^

n = 1, other treatments^67^

n = 2, duplicate abstracts^38, 39^

**Table S3:** Detailed inclusion and exclusion criteria for the studies included in this systematic review and meta-analysis.

| No | Author | Time point at which primary outcomes were measured | Inclusion criteria | Exclusion criteria |
| --- | --- | --- | --- | --- |
| 1 | Lynch et al^68^ | 56 days after last infusion | - Patients must have been diagnosed with PSC according to internationally guidelines, - have received a minimum of 3 doses of vedolizumab for their IBD, have baseline (pre-vedolizumab) and follow-up blood tests including liver biochemistry, - have commenced vedolizumab with their native liver still in situ and - received vedolizumab according to the usual dosing schedule as licensed. | NA |
| 2 | Caron B et al^69^ | Week 30 | - Consecutive adult patients with both IBD and PSC, who received at least one injection of vedolizumab, were included. - Diagnosis of PSC was confirmed by two of the following criteria after exclusion of other cholestatic disorders: - [1] chronic cholestatic liver disease of at least 6 months duration. - [2] intrahepatic and/or extrahepatic biliary duct changes, such as beading or narrowing consistent with PSC as demonstrated by magnetic resonance cholangiography; and - [3] liver biopsy compatible with the diagnosis of PSC. | - Age < 18 years - PSC without IBD, inactive CD (Harvey Bradshaw Index<4) or UC (partial MAYO score<3) and - Pregnancy or lactation |
| 3 | Christensen B et al^70^ | Week 30 | - Electronic medical records at participating sites were reviewed for adult patients with an established diagnosis of concurrent IBD and PSC (IBD-PSC) based on clinical, biochemical, imaging and endoscopic information and who had been initiated on vedolizumab between June 2014 and January 2016. | - NA |
| 4 | Tse C et al^71^ | 6-8 month | - Patients with IBD with concurrent PSC treated with infliximab, adalimumab, or vedolizumab between June 2002 and October 2017. - Of the 75 study patients, 63 received 1 biologic, 11 received 2 biologics, and 1 received 3 biologics; - patients who received >1 biologics were included more than once to reflect the effects on LFT’s with each biological use (regarded as an individual case). | - prior liver transplantation, - duration of ≤6 months of biological therapy, - absence of elevated LFTs ≤3 months prior to biological initiation, - age <18 years |
| 5 | Hedin C et al^72^ | Week 12 | - Patients were included if they had received at least 2 doses of anti-TNF and had baseline blood tests (not >2 months before drug initiation and <7 days after drug initiation). - Patients with liver transplantation before anti-TNF initiation were considered separately. | - Patients with insufficient data were excluded. |
| 6 | Hommes D et al^73^ | Week 52 | - Patients had to be at least 18 years of age and - have had a baseline alkaline phosphatase level at least 2 times the upper limit of normal. - Treatment with UDCA was allowed, if treatment was stable beginning 1 month before study start and continuing through the end of the study. | - Patients with a Crohn’s disease activity index greater than 350, evidence of secondary sclerosing cholangitis, or with other liver disease were not eligible. - Previous treatment with infliximab, - patients who received any other agent targeted at TNF reduction within 3 months of screening, or - who required immunosuppressive or anti-inflammatory medication other than mesalazine derivatives were excluded from study. |

LFT, liver function test; PSC, primary sclerosing cholangitis; NA, not applicable; UC, ulcerative colitis; CD, Crohn’s disease, IBD, inflammatory bowel disease; TNF’ tumour necrosis factor; UDCA, Ursodeoxycholic acid.

**Table S4:** Summary of findings reported in the systematic review and meta-analysis.

|  | **Studies, n** | **Patients with PSC and IBD, n** | **Change in liver enzymes post treatment with biologic therapy, values expressed as SMD (95% CI)** | **Assessment of heterogeneity between studies** |
| --- | --- | --- | --- | --- |
| **Studies assessing change in ALP** | 6 | 355 | 0.05 (-0.07-0.17), p=0.43 | I^2^=76.1, p<0.01 |
| **Studies assessing change in total bilirubin** | 5 | 312 | 0.2 (0.05-0.35), p=0.01 | I^2^=30.5, p=0.184 |
| **Studies assessing change in AST** | 4 | 256 | 0.15 (-0.03-0.33), p=0.09 | I^2^=35.02, p=0.17 |
| **Studies assessing change in ALT** | 4 | 257 | 0.06 (-0.09-0.22), p=0.44 | I^2^=27.6, p=0.23 |
| **Studies assessing change in UC response score** | 2 | 99 | -0.62 (-0.88, -0.36), p<0.01 | I^2^=0, p=0.94 |
|  | **Studies, n** | **Patients with PSC and IBD, n** | **Proportion of PSC-IBD patients treated with biologics, values expressed as % (95% CI)** | **Assessment of heterogeneity between studies** |
| **Studies assessing proportion of patients who developed adverse events** | 4 | 392 | 17.6 (95%CI:13.0-23.5) | I^2^=79.7, p<0.01 |
| **Studies assessing proportion of patients who had loss or response to biologic therapy** | 4 | 368 | 29.9 (25.2-34.8) |  |
| **Studies assessing proportion of patients who had endoscopic improvement in their colitis** | 4 | 216 | 31.2 (23.8-39.7) | I^2^=84.5, p<0.01 |
| **Studies assessing proportion of patients who had clinical response of their associated colitis** | 3 | 183 | 47% (39.6-54.5) |  |

PSC, Primary Sclerosing Cholangitis; UC, ulcerative colitis; IBD, Inflammatory bowel disease; CI, confidence interval; n, number;

SMD, standardized mean difference; ALP, Alkaline phosphatase enzyme; AST Aspartate transaminase enzyme; ALT, Alanine transaminase enzyme.

**Table S5:** Joanna Briggs Institute (JBI) Critical Appraisal Tools for quality assessment of the studies included in the meta-analysis.

|  |  | 1. Was the sample frame appropriate to address the target population? | 2. Were study participants sampled in an appropriate way? | 3. Was the sample size adequate? | 4. Were the study subjects and the setting described in detail? | 5. Was the data analysis conducted with sufficient coverage of the identified sample? | 6. Were valid methods used for the identification of the condition? | 7. Was the condition measured in a standard, reliable way for all participants? | 8. Was there appropriate statistical analysis? | 9. Was the response rate adequate, and if not, was the low response rate managed appropriately? | Risk of bias |
| --- | --- | --- | --- | --- | --- | --- | --- | --- | --- | --- | --- |
| 1 | Lynch K et al^68^ | Yes | Unclear | Yes | Yes | Yes | Yes | Yes | Yes | NA | Low |
| 2 | Caron B et al^69^ | Yes | Yes | Yes | Yes | Yes | Yes | Yes | Yes | NA | Low |
| 3 | Christensen B et al^70^ | Yes | Yes | Yes | Yes | Yes | Yes | Yes | Yes | NA | Low |
| 4 | Tse C et al^71^ | Yes | Yes | Yes | Yes | Yes | Yes | Yes | Yes | NA | Low |
| 5 | Hedin C et al^72^ | Yes | Unclear | Yes | No | No | Yes | Yes | Yes | NA | Moderate |
| 6 | Hommes D et al^73^ | No | Yes | Yes | Yes | Yes | Yes | Yes | Yes | Unclear | Low |

NA: Not applicable; A: abstract.

**References:**

1. Schroeder KW, Tremaine WJ, Ilstrup DM. Coated Oral 5-Aminosalicylic Acid Therapy for Mildly to Moderately Active Ulcerative Colitis. New England Journal of Medicine 1987;317:1625-1629.

2. Simon PLT, Dan S, Piotr K, et al. Developing an instrument to assess the endoscopic severity of ulcerative colitis: the Ulcerative Colitis Endoscopic Index of Severity (UCEIS). Gut 2012;61:535.

3. Daperno M, D'Haens G, Van Assche G, et al. Development and validation of a new, simplified endoscopic activity score for Crohn's disease: the SES-CD. Gastrointestinal Endoscopy 2004;60:505-512.

4. Lewis JD, Chuai S, Nessel L, et al. Use of the noninvasive components of the Mayo score to assess clinical response in ulcerative colitis. Inflamm Bowel Dis 2008;14:1660-6.

5. Harvey RF, Bradshaw MJ. Measuring Crohn's disease activity. Lancet 1980;1:1134-5.

6. Best WR, Becktel JM, Singleton JW, et al. Development of a Crohn's disease activity index. National Cooperative Crohn's Disease Study. Gastroenterology 1976;70:439-44.

7. Munn Z, Moola S, Lisy K, et al. Methodological guidance for systematic reviews of observational epidemiological studies reporting prevalence and cumulative incidence data. Int J Evid Based Healthc 2015;13:147-53.

8. Hozo SP, Djulbegovic B, Hozo I. Estimating the mean and variance from the median, range, and the size of a sample. BMC Medical Research Methodology 2005;5:13.

9. Wan X, Wang W, Liu J, et al. Estimating the sample mean and standard deviation from the sample size, median, range and/or interquartile range. BMC Med Res Methodol 2014;14:135.

10. Bowlus CL, Arrivé L, Bergquist A, et al. AASLD practice guidance on primary sclerosing cholangitis and cholangiocarcinoma. Hepatology 2023;77.

11. Chazouilleres O, Beuers U, Bergquist A, et al. EASL Clinical Practice Guidelines on sclerosing cholangitis. Journal of hepatology 2022;77:761-806.

12. Biscaglia G, Piazzolla M, Cocomazzi F, et al. Landmarks for dual biological therapy in inflammatory bowel disease: lesson from two case reports of vedolizumab in combination with ustekinumab. Eur J Gastroenterol Hepatol 2020;32:1579-1582.

13. Coletta M, Paroni M, Caprioli F. Successful Treatment With Vedolizumab in a Patient With Chronic Refractory Pouchitis and Primary Sclerosing Cholangitis. J Crohns Colitis 2017;11:1507-1508.

14. Damas OM, Estes D, Polanco NAP, et al. Treatment of Inflammatory Bowel Disease With Vedolizumab for Patients With Coexisting Primary Sclerosing Cholangitis: Report of a Nested Case-Control Study. GASTROENTEROLOGY 2016;150:S1074-S1074.

15. Del Ross T, Ruffatti A, Floreani A, et al. The efficacy of adalimumab in psoriatic arthritis concomitant to overlapping primary biliary cholangitis and primary sclerosing cholangitis: a case report. BMC Musculoskelet Disord 2016;17:485.

16. Duca I, Ramirez de la Piscina P, Estrada S, et al. Steroid-refractory ulcerative colitis and associated primary sclerosing cholangitis treated with infliximab. World J Gastroenterol 2013;19:590-3.

17. Varkas G, Thevissen K, De Brabanter G, et al. An induction or flare of arthritis and/or sacroiliitis by vedolizumab in inflammatory bowel disease: a case series. Ann Rheum Dis 2017;76:878-881.

18. Westerveld D, Grajo J, Beattie L, et al. Vedolizumab: a novel medical intervention in the treatment of primary sclerosing cholangitis. BMJ Case Rep 2017;2017.

19. Doherty J, Buckley M, Cullen G, et al. Vedolizumab: Effects on liver function in an IBD and IBD/PSC cohort. JOURNAL OF CROHNS & COLITIS 2018;12:S402-S402.

20. Franceschet I, Cazzagon N, Del Ross T, et al. Primary sclerosing cholangitis associated with inflammatory bowel disease: an observational study in a Southern Europe population focusing on new therapeutic options. Eur J Gastroenterol Hepatol 2016;28:508-13.

21. Lim TY, Pavlidis P, Pirani T, et al. Vedolizumab in primary and autoimmune sclerosing cholangitis associated inflammatory bowel disease pre and post liver transplantation: a case series

GUT 2016;65:A89-A89.

22. Angelison L, Almer S, Davidsdottir L, et al. Short and long-term efficacy of adalimumab in ulcerative colitis: a real-life study. Scand J Gastroenterol 2020;55:154-162.

23. Barrie A, Regueiro M. Biologic therapy in the management of extraintestinal manifestations of inflammatory bowel disease. Inflammatory bowel diseases 2007;13:1424-1429.

24. Benjamin C, Tom B, Jimmy KL. Vedolizumab in the treatment of inflammatory bowel disease: evolving paradigms. Drugs in Context 2020;9:1-15.

25. Crispino F, Grova M, Bruno EM, et al. Spondyloarthropathy in Inflammatory Bowel Disease: From Pathophysiology to Pharmacological Targets. Drugs 2022;82:1151-1163.

26. Guidi L, Pugliese D, Armuzzi A. Update on the management of inflammatory bowel disease: specific role of adalimumab. Clin Exp Gastroenterol 2011;4:163-72.

27. Lal S, Steinhart AH. Infliximab for ulcerative colitis following liver transplantation. European Journal of Gastroenterology & Hepatology 2007;19:277-280.

28. Feagan BG, Sandborn WJ, Colombel JF, et al. Incidence of Arthritis/Arthralgia in Inflammatory Bowel Disease with Long-term Vedolizumab Treatment: Post Hoc Analyses of the GEMINI Trials. J Crohns Colitis 2019;13:50-57.

29. Franceschet I, Cazzagon N, Floreani A. Effectiveness of adalimumab for patients with primary sclerosing cholangitis associated with inflammatory bowel disease. JOURNAL OF HEPATOLOGY 2015;62:S799-S799.

30. Lynch K, Chapman R, Klenerman P, et al. Vedolizumab alters the distribution of gut-homing T cells within the human body in patients with inflammatory bowel disease, including those with primary sclerosing cholangitis. JOURNAL OF GASTROENTEROLOGY AND HEPATOLOGY 2019;34:113-113.

31. Schregel I, Ramos GP, Ioannou S, et al. Evaluation of Tofacitinib in Primary Sclerosing Cholangitis and Associated Colitis: A Multicenter, Retrospective Study. Clin Gastroenterol Hepatol 2023.

32. Travis SP, Feagan BG, Peyrin-Biroulet L, et al. Effect of adalimumab on extraintestinal manifestations among patients with ulcerative colitis in a clinical practice setting: results from inspirada. GASTROENTEROLOGY 2017;152:S743-S743.

33. Tse CS, Loftus EV, Raffals LH, et al. Vedolizumab is not associated with increased infection risk in immunosuppressed liver transplant recipients with inflammatory bowel disease and primary sclerosing cholangitis. GASTROENTEROLOGY 2019;156:S636-S636.

34. Christensen B, Micic D, Gibson PR, et al. Sa1950 - Vedolizumab is Safe and Effective for IBD, but has no Effect on Liver Biochemistry in Patients with Concurrent PSC. Gastroenterology 2017;152:S405-S405.

35. Gold S, Cohen-Mekelburg SA, Schneider Y, et al. Mo1899 - Joint Pain in Patients with Inflammatory Bowel Disease Treated with Vedolizumab: An Extra-Intestinal Manifestation or a Side Effect of a Gut-Specific Therapy? Gastroenterology 2018;154:S.

36. Kim SB, Diaz LI, Calmet F, et al. Sa1696 - Vedolizumab Induces De Novo Extraintestinal Manifestations in Patients with Inflammatory Bowel Disease. Gastroenterology 2018;154:S.

37. Tse CS, Raffals LH, Loftus EV, et al. Sa1430 - Effect of Vedolizumab, Infliximab, and Adalimumab on Biliary Inflammation in Individuals with Primary Sclerosing Cholangitis and Inflammatory Bowel Disease. Gastroenterology 2018;154:S.

38. Williamson KD, Lytvyak E, de Krijger M, et al. 558 - International Experience of Vedolizumab in Primary Sclerosing Cholangitis and Inflammatory Bowel Disease. Gastroenterology 2018;154:S.

39. Williamson KD, Slevin S, Willberg C, et al. SAT-370 - Clinical and translational outcomes in patients with primary sclerosing cholangitis and inflammatory bowel disease receiving vedolizumab. Journal of Hepatology 2017;66:S544-S545.

40. Williamson KD, Slevin S, Willberg C, et al. Mo1447 - Clinical and Translational Outcomes in Patients with Primary Sclerosing Cholangitis and Inflammatory Bowel Disease Receiving Vedolizumab. Gastroenterology 2017;152:S1186-S1187.

41. Aasem Abu S, Shai C, Yana K, et al. Crohn’s Disease with Atypical Extra-Intestinal Manifestations Developing Under Treatment with Vedolizumab. European Journal of Case Reports in Internal Medicine 2021.

42. Barclay ML, Stamp LK. Editorial: vedolizumab as a treatment and cause of extra-intestinal manifestations of inflammatory bowel disease. Alimentary pharmacology & therapeutics 2018;47:535-536.

43. Barreiro-de-Acosta M, Lorenzo A, Domínguez-Muñoz JE. Efficacy of adalimumab for the treatment of extraintestinal manifestations of Crohn's disease. Revista Espanola de Enfermedades Digestivas 2012;104:468-472.

44. De Galan C, Truyens M, Peeters H, et al. The Impact of Vedolizumab and Ustekinumab on Articular Extra-Intestinal Manifestations in Inflammatory Bowel Disease Patients: A Real-Life Multicentre Cohort Study. Journal of Crohn's & colitis 2022;16:1676-1686.

45. Diaz LI, Keihanian T, Schwartz I, et al. Vedolizumab-Induced De Novo Extraintestinal Manifestations. Gastroenterology & Hepatology 2020;16:74-81.

46. Dimopoulos C, Hung K, Proctor D, et al. Effects of vedolizumab on extraintestinal manifestations in inflammatory bowel disease. GASTROENTEROLOGY 2020;158:S32-S33.

47. Dubinsky M, Cross R, Jr., Sandborn W, et al. The Incidence of Extraintestinal Manifestations in Patients with Inflammatory Bowel Disease Treated with Vedolizumab and Anti-TNF Therapies. INFLAMMATORY BOWEL DISEASES 2017;23:S17-S18.

48. Eksteen B, Heatherington J, Oshiomogho JI, et al. Efficacy and Safety of Induction Dosing of Vedolizumab for Reducing Biliary Inflammation in Primary Sclerosing Cholangitis (PSC) in Individuals With Inflammatory Bowel Disease. GASTROENTEROLOGY 2016;150:S1268-S1268.

49. Eksteen B, Heatherington J, Oshiomogo J, et al. RETRACTED: Efficacy and Safety of Induction Dosing of Vedolizumab for Reducing Biliary Inflammation in Primary Sclerosing Cholangitis (Psc) in Individuals with Inflammatory Bowel Disease. Journal of hepatology 2016;64:S199.

50. Gold S, Cohen-Mekelburg SA, Schneider Y, et al. Joint pain in patients with inflammatory bowel disease treated with vedolizumab: an extra-intestinal manifestation or a side effect of a gut-specific therapy? GASTROENTEROLOGY 2018;154:S844-S844.

51. Halina C-L, Agata M, Maria K-M, et al. Characteristics of patients with moderate-to-severe ulcerative colitis treated with vedolizumab: results from a Polish multicenter, prospective, observational real-life study (the POLONEZ study). Therapeutic Advances in Gastroenterology 2021;14.

52. Hanzel J, Ma C, Casteele NV, et al. Vedolizumab and Extraintestinal Manifestations in Inflammatory Bowel Disease. Drugs 2021;81:333-347.

53. Herrlinger KR, Stange EF. Twenty-five years of biologicals in IBD: What´s all the hype about? Journal of internal medicine 2021;290:806-825.

54. Katsanos KH, Fousekis FS, Armuzzi A. The role of ustekinumab and vedolizumab in management of extra intestinal manifestations in inflammatory bowel disease. Digestive and liver disease : official journal of the Italian Society of Gastroenterology and the Italian Association for the Study of the Liver 2022.

55. Kim SB, Diaz LI, Calmet F, et al. Vedolizumab induces de novo extraintestinal manifestations in patients with inflammatory bowel disease

GASTROENTEROLOGY 2018;154:S360-S360.

56. Kopylov U, Burisch J, Ben-Horin S, et al. A retrospective analysis of the efficacy of vedolizumab on extra-intestinal manifestations in patients with inflammatory bowel disease across five European countries. JOURNAL OF CROHNS & COLITIS 2021;15:S412-S413.

57. Livne-Margolin M, Ling D, Attia-Konyo S, et al. Ustekinumab and vedolizumab for extraintestinal manifestations in inflammatory bowel disease - a retrospective study. Digestive and liver disease : official journal of the Italian Society of Gastroenterology and the Italian Association for the Study of the Liver 2022.

58. Mosli M, Croome K, Qumosani K, et al. The Effect of Liver Transplantation for Primary Sclerosing Cholangitis on Disease Activity in Patients with Inflammatory Bowel Disease. Gastroenterology & Hepatology 2013;9:434-441.

59. Patel RJ, Grimes I. Vedolizumab in Inflammatory Bowel Disease: A Retrospective Review of Clinical Efficacy, Extra-intestinal Manifestations and Adverse Reactions. AMERICAN JOURNAL OF GASTROENTEROLOGY 2016;111:S317-S318.

60. Perez Galindo P, Gisbert JP, Carrillo-Palau M, et al. Influence of vedolizumab on extraintestinal manifestations in Inflammatory Bowel Disease: a nationwide multicenter study of the GETECCU Eneida registry. JOURNAL OF CROHNS & COLITIS 2021;15:S308-S309.

61. Ramos GP, Dimopoulos C, McDonald NM, et al. The Impact of Vedolizumab on Pre-Existing Extraintestinal Manifestations of Inflammatory Bowel Disease: A Multicenter Study. Inflammatory Bowel Diseases 2021;27:1270-1276.

62. Tadbiri S, Grimaud J-c, Peyrin-Biroulet L, et al. Sa1927 - Efficacy of Vedolizumab on Extraintestinal Manifestation in Patients with Inflammatory Bowel Diseases: A Post-Hoc Analysis of the Observ-IBD Cohort of the Getaid. Gastroenterology 2017;152:S396-S396.

63. Tadbiri S, Peyrin-Biroulet L, Serrero M, et al. Impact of vedolizumab therapy on extra-intestinal manifestations in patients with inflammatory bowel disease: a multicentre cohort study nested in the OBSERV-IBD cohort. ALIMENTARY PHARMACOLOGY & THERAPEUTICS 2018;47:485-493.

64. Truyens M, Geldof J, Dewitte G, et al. The impact of vedolizumab on extra-intestinal manifestations in inflammatory bowel disease patients: A real-life experience of a single-centre cohort. JOURNAL OF CROHNS & COLITIS 2020;14:S546-S547.

65. Tzadok R, Fliss-Isakov N, Cohen NA, et al. Serum vedolizumab trough levels are associated with remission rate but not with extra-intestinal manifestations in IBD patients. JOURNAL OF CROHNS & COLITIS 2021;15:S441-S442.

66. Epstein MP, Kaplan MM. A pilot study of etanercept in the treatment of primary sclerosing cholangitis. Dig Dis Sci 2004;49:1-4.

67. Bharucha AE, Jorgensen R, Lichtman SN, et al. A pilot study of pentoxifylline for the treatment of primary sclerosing cholangitis. Am J Gastroenterol 2000;95:2338-42.

68. Lynch KD, Chapman RW, Keshav S, et al. Effects of Vedolizumab in Patients With Primary Sclerosing Cholangitis and Inflammatory Bowel Diseases. Clin Gastroenterol Hepatol 2020;18:179-187.e6.

69. Caron B, Peyrin-Biroulet L, Pariente B, et al. Vedolizumab Therapy is Ineffective for Primary Sclerosing Cholangitis in Patients With Inflammatory Bowel Disease: A GETAID Multicentre Cohort Study. J Crohns Colitis 2019;13:1239-1247.

70. Christensen B, Micic D, Gibson PR, et al. Vedolizumab in patients with concurrent primary sclerosing cholangitis and inflammatory bowel disease does not improve liver biochemistry but is safe and effective for the bowel disease. Aliment Pharmacol Ther 2018;47:753-762.

71. Tse CS, Loftus Jr EV, Raffals LE, et al. Effects of vedolizumab, adalimumab and infliximab on biliary inflammation in individuals with primary sclerosing cholangitis and inflammatory bowel disease. Alimentary Pharmacology & Therapeutics 2018;48:190-195.

72. Hedin CRH, Sado G, Ndegwa N, et al. Effects of Tumor Necrosis Factor Antagonists in Patients With Primary Sclerosing Cholangitis. Clin Gastroenterol Hepatol 2020;18:2295-2304.e2.

73. Hommes D, Erkelens W, Ponsioen C, et al. A Double-blind, Placebo-controlled, Randomized Study of Infliximab in Primary Sclerosing Cholangitis. Journal of Clinical Gastroenterology 2008;42.
